# Pharmacokinetics of gefitinib in elderly patients with *EGFR*-mutated advanced non-small cell lung cancer: a prospective study

**DOI:** 10.1186/s12890-022-02249-8

**Published:** 2022-11-30

**Authors:** Yuta Nio, Hiroo Ishida, Natsumi Matsumoto, Sojiro Kusumoto, Yutaro Kubota, Takuya Tsunoda, Yasutsuna Sasaki, Ken-ichi Fujita

**Affiliations:** 1 Department of Hospital Pharmaceutics, Showa University School of Pharmacy, 1-5-8 Hatanodai, Shinagawa-ku, Tokyo, 142-8555 Japan; 2 Division of Medical Oncology, Department of Medicine, Showa University School of Medicine, 1-5-8 Hatanodai, Shinagawa-ku, Tokyo, 142-8555 Japan; 3 Division of Cancer Genome and Pharmacotherapy, Department of Clinical Pharmacy, Showa University School of Pharmacy, 1-5-8 Hatanodai, Shinagawa-ku, Tokyo, 142-8555 Japan; 4 Division of Respirology and Allergology, Department of Medicine, Showa University School of Medicine, 1-5-8 Hatanodai, Shinagawa-ku, Tokyo, 142-8555 Japan

**Keywords:** Gefitinib, Pharmacokinetics, Adverse events, Elderly patients

## Abstract

**Background:**

Gefitinib is recommended as a first-line treatment option for elderly patients with non-small cell lung cancer (NSCLC). Because no pharmacokinetics of gefitinib have been examined, we prospectively assessed the pharmacokinetics of gefitinib in patients with epidermal growth factor receptor gene-mutated advanced NSCLC who were 75 years or older.

**Methods:**

Gefitinib was orally administered once daily at a dose of 250 mg. The concentrations of gefitinib and its major metabolite *O*-desmethyl gefitinib in plasma were measured by high-performance liquid chromatography. The area under the plasma concentration–time curve from time 0 to 48 h (AUC_0–48_) was calculated. Polymorphisms in *CYP3A5*, *CYP2D6*, *ABCG2*, *ABCB1*, and *OATP1B1* were analyzed by direct sequencing.

**Results:**

Eighteen patients with a median age of 80.5 years (range, 75–89) with adequate liver and kidney functions were examined. AUC_0–48_ values of gefitinib and *O*-desmethyl gefitinib in this population were 9.49 ± 3.5 and 10.6 ± 14 µM h, respectively. Compared to the gefitinib pharmacokinetics observed in a previous phase I study in Japan, systemic exposure to gefitinib in elderly patients was slightly higher than that in younger patients. Three patients experienced grade 3 diarrhea, increases in alanine aminotransferase, and aspartate aminotransferase levels 30 days after starting gefitinib treatment. The *CYP2D6* genotype was associated with CYP2D6-mediated metabolism of gefitinib to *O*-desmethyl gefitinib.

**Conclusions:**

We demonstrated for the first time the systemic exposure to gefitinib in elderly patients with NSCLC.

*Trial registration*: The study was registered with the University Hospital Medical Information Network-Clinical Trials Registry Japan (UMIN000026409) on November 8, 2013.

**Supplementary Information:**

The online version contains supplementary material available at 10.1186/s12890-022-02249-8.

## Background

Gefitinib is a selective tyrosine kinase inhibitor (TKI) of the epidermal growth factor receptor (EGFR). Based on phase II studies in previously treated patients with non-small cell lung cancer (NSCLC) that demonstrated objective response rates of ~ 18% with manageable adverse events [1, 2], gefitinib was approved in Japan for the treatment of patients with advanced NSCLC. Subsequently, somatic mutations in the tyrosine kinase domain of the *EGFR* gene were identified in patients with gefitinib-responsive lung cancer, whereas no such mutations were observed in patients with no response. These mutations in exon 19 or 21 have been recognized as a biomarker for the efficacy of gefitinib therapy [3, 4]. Two phase III studies with previously untreated patients with NSCLC who harbored *EGFR*-activating mutations demonstrated significant improvement in survival among gefitinib groups compared with platinum-based chemotherapy groups, and these findings established gefitinib as a standard treatment for *EGFR*-mutated NSCLC [5, 6].

Later, a second generation irreversible TKI of EGFR, afatinib, became a novel standard treatment option based on the results of a phase III study that showed significant improvement in overall survival with afatinib compared with platinum-based chemotherapy in patients with *EGFR* mutations [7]. However, afatinib was intolerable among patients who were elderly or had a poor performance status, because the TKI induced more frequent and severe adverse events, especially diarrhea and skin toxicity, than gefitinib [8, 9]. Because a phase II study in elderly advanced patients with NSCLC with *EGFR* mutations demonstrated similar efficacy and toxicity of gefitinib [10] as previous studies of gefitinib including younger patients [5, 6], gefitinib has been recommended as a first-line treatment option for elderly patients. However, other studies on the efficacy and safety of gefitinib are scant. Nonetheless, the number of elderly patients with cancer is increasing in Japan [11], which implies an increase in elderly patients with advanced NSCLC who are candidates for treatment with gefitinib. Furthermore, the pharmacokinetics of gefitinib and one of its major metabolites, *O*-desmethyl gefitinib, have not been evaluated in this population, although gefitinib pharmacokinetics are associated with toxicities induced by this drug [12].

Gefitinib is extensively metabolized by CYP3A4, with CYP3A5 and CYP2D6 having relatively minor roles [13]. The major human plasma metabolite is formed predominantly by CYP2D6 in human liver microsomes [13]. Gefitinib is also reportedly a substrate of human ATP-binding cassette transporters, ABCG2 (breast cancer resistance protein), and ABCB1 (P-glycoprotein) [12]. Some polymorphisms in the genes of these drug-metabolizing enzymes and transporters are associated with gefitinib pharmacokinetics and toxicities induced by the TKI [12].

Given this background information, we prospectively assessed the pharmacokinetics of gefitinib in patients with *EGFR*-mutated advanced NSCLC who were 75 years or older. We further examined the effects of genetic polymorphisms in genes encoding drug-metabolizing enzymes and transporters on pharmacokinetics of gefitinib.

## Methods

### Chemicals

Gefitinib was purchased from LC Laboratories (Woburn, MA, USA), and *O*-desmethyl gefitinib was obtained from Toronto Research Chemicals (Toronto, Canada). All chemicals and solvents were of the highest grade commercially available.

### Study design

This was a prospective study to assess associations of the pharmacokinetics, pharmacogenetics, and toxicity of gefitinib in *EGFR* mutated patients who were 75 years or older, had not previously received EGFR-TKI and were candidate for gefitinib treatment at Showa University Hospital. Our objective was to examine the pharmacokinetics of gefitinib and its metabolites, and the effects of polymorphisms in genes encoding factors related to gefitinib pharmacokinetics of the pharmacokinetics. The study protocol was approved by the Institutional Review Board of Showa University. All patients provided written informed consent to use their peripheral blood samples and medical information for research purposes. The study was registered with the University Hospital Medical Information Network-Clinical Trials Registry Japan (UMIN000026409).

### Patients

Eligible patients were 75 years or older. Patients had histologically or cytologically confirmed unresectable advanced or recurrent NSCLC harboring the *EGFR* mutation. Eligible patients also had an Eastern Cooperative Oncology Group performance status of 0–2, a life expectancy of 2 months or longer, and no history of chemotherapy within 2 weeks. All patients were confirmed to have adequate bone marrow function (neutrophil count ≥ 1500/μL; platelet count ≥ 100,000/μL; hemoglobin level ≥ 8.0 g/dL) and liver function (total bilirubin level ≤ 2.0 mg/dL; alanine transaminase [ALT] and aspartate transaminase [AST] level ≤ 2.0 × the upper limit of normal) within 14 days of the initiation of gefitinib treatment. Patients were excluded if they had taken medication that affects CYP3A4, such as proton-pump inhibitors and histamine H_2_ receptor antagonists.

### Treatment

Gefitinib (250 mg; Iressa; AstraZeneca, Osaka, Japan) was orally administered once daily after breakfast. Gefitinib on day 2 was skipped for pharmacokinetic analysis of the first gefitinib dose. The treatment was continued until disease progression, unacceptable toxicity, or patient refusal.

### Blood sampling for pharmacokinetic analysis

Blood samples for pharmacokinetic analysis of gefitinib and *O*-desmethyl gefitinib were obtained on the first day of administration. Blood samples were taken immediately before gefitinib administration and at 1, 2, 4, 6, 8, 24, and 48 h after gefitinib administration. The samples were centrifuged immediately and stored at − 80 °C until analysis.

### Determination of gefitinib and *O*-desmethyl gefitinib concentrations

Plasma concentrations of gefitinib and *O*-desmethyl gefitinib were measured using reverse-phase high-performance liquid chromatography as described previously [14]. The quantification limit for both gefitinib and *O*-desmethyl gefitinib was 0.04 μM. The respective intra- and inter-assay coefficients of variation for gefitinib and *O*-desmethyl gefitinib at the quantification limits were within 20%. Differences in measured- and spiked-concentrations of gefitinib and *O*-desmethyl gefitinib at the quantification limits were within 20%.

### Pharmacokinetic parameters

The plasma concentration–time data of gefitinib and *O*-desmethyl gefitinib were analyzed using a standard non-compartmental method with WinNonlin, version 8.3 software (Pharsight, Mountain View, CA, USA). The area under the plasma concentration–time curve (AUC) of gefitinib and *O*-desmethyl gefitinib from time zero to the last sampling time was calculated using the linear trapezoidal rule (up to the peak plasma concentration) and linear-log trapezoidal rule (up to the last quantifiable concentration). Times to the maximum plasma concentration (T_max_), maximum plasma concentration (C_max_), and elimination half-life (t_1/2_) were also determined. Gefitinib oral clearance (CL/F, L/h) was obtained by dividing the single gefitinib dose (μmol/body, calculated based on the molecular weight, 446.9) by the AUC, with extrapolation to infinity (dose/AUC).

### Evaluation of toxicity

Toxicities in our patients were observed over 30 days after initiation of gefitinib treatment. Clinical and laboratory adverse events were classified according to the National Cancer Institute Common Terminology Criteria for adverse events version 4.0. If adverse events advanced greater than grade 3 or dose reduction was necessary according to the medical oncologist, after the adverse events improved to grade 1, patients were administered 250 mg of gefitinib every other day.

### Genotyping

Genomic DNA was extracted from 200 µL of peripheral blood stored at − 80 °C using the QIAamp Blood Kit (QIAGEN GmbH, Hilden, Germany).

The gene fragment of *CYP3A5* containing 6986T > C (rs776746, CYP3A5*3) was amplified by polymerase chain reaction (PCR) with the primers established previously [15]. PCR was performed in a total volume of 50 μL in the presence of 100 ng of genomic DNA, 1 × PCR buffer, 1 mM MgCl_2_, 0.2 mM dNTPs, 0.1 μM of each primer, and 1.25 U of the AmpliTaq Gold DNA polymerase (Applied Biosystems, Waltham, MA, USA). Cycling conditions were follows: initial denaturation at 94 °C for 15 min was followed by 30 cycles of 30 s at 94 °C, 1 min at 55 °C, and 2 min at 72 °C, as well as a final extension period of 7 min at 72 °C.

The *CYP2D6* gene fragment containing 100C > T (rs1065852, P34S, CYP2D6*10) was amplified by PCR with the primers described elsewhere [16]. A total of 100 ng genomic DNA samples were added to the PCR mixtures (50 μL), consisting of 1 × PCR buffer, 2 mM MgCl_2_, 0.2 mM dNTPs, 0.2 μM of each primer, and 1.25 U of the AmpliTaq Gold DNA polymerase. The cycling protocol consisted of denaturation at 94 °C for 15 min, followed by 30 cycles of 94 °C for 30 s, 60 °C for 1 min, 72 °C for 1 min, and a final extension at 72 °C for 10 min to hold.

Gene fragments containing *ABCG2* 421C > A (rs2231142, Q141K), *ABCB1* 1236C > T (rs1128503, G412G), 2677G > T/A (rs2032582, A893T/S), 3435C > T (rs1045642, I1145I), the organic anion transporting polypeptide (*OATP1B1*) 388A > G (rs2306283, N130D), and 521T > C (rs4149056, V174A) were amplified by PCR using the methods described previously [17, 18].

All aforementioned PCR products were purified and sequenced directly.

For detection of CYP2D6*5, PCR was performed using Hersberger et al.’s methods [19].

Allele and genotype frequencies for each polymorphic allele genes were determined using SNPAlyze 8.1.1 (Dynacom, Yokohama, Japan). The significance of deviations from the Hardy–Weinberg equilibrium was tested with the program SNPAlyze 8.1.1. Analyses of diplotype configurations (combinations of haplotypes) analyses in *ABCB1* gene (1236C > T, 2677G > T/A, and 3435C > T) and *OATP1B1* gene (388G > A and 521T > C) were also performed using an expectation–maximization-based algorithm using SNPAlyze 8.1.1.

### Statistical analysis

Associations between genotypes or diplotypes and AUC values were analyzed using the Wilcoxon or Kruskal–Wallis test. Because these analyses were exploratory, criterion of statistical significance was not set. All analyses were performed using the JMP software, version 16.0.0 (SAS Institute, Cary, NC, USA).

## Results

### Patient characteristics

Eighteen patients with adenocarcinoma aged 75 years or older were enrolled in this study between January 2014 and April 2018. Histology results of all patients indicated adenocarcinoma. Table 1 shows the characteristics of the patients. All patients showed liver and kidney functions that met the eligibility criteria (Additional file 1: Table S1).

**Table 1:** Patient characteristics

	N = 18
Age (years)	80.5 (75–89)^a^
Sex	
Male/female	6/12
Performance status	
0/1/2/3	1/9/5/3
Body weight (kg)	47.7 (38.3–65.7)^a^
Serum albumin level (g/dL)	3.6 (1.6–4.5)^a^
Total bilirubin level (mg/dL)	0.6 (0.4–1.0)^a^
Serum creatinine level (mg/dL)	0.73 (0.38–1.14)^a^
eGFR (mL/min)	60.2 (48.5–115)^a^
*EGFR* mutation	
Exon 19/exon 21	8/10

*eGFR* estimated glomerular filtration rate, *EGFR* epidermal growth factor receptor

^a^Data represented the median (range)

### Pharmacokinetics

**Fig. 1:**
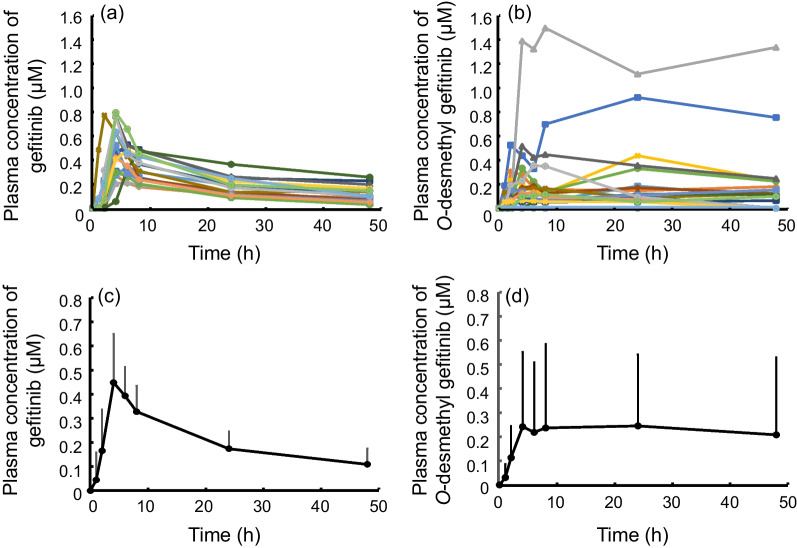
Pharmacokinetic profiles of gefitinib (**a**, **c**) and *O*-desmethyl gefitinib (**b**, **d**) in elderly patients with NSCLC. N = 18. **a** and **b** depict the individual plasma concentration profiles of gefitinib and *O*-desmethyl gefitinib, respectively. **c** and **d** represent the average plasma concentration profiles of gefitinib and *O*-desmethyl gefitinib, respectively. Each data point indicates the mean ± SD. *NSCLC* non-small cell lung cancer, *SD* standard deviation

Pharmacokinetic profiles and pharmacokinetic parameters of gefitinib and its metabolite *O*-desmethyl gefitinib are shown in Fig. 1 and Table 2, respectively. Multiple peaks in plasma concentration profiles of *O*-desmethyl gefitinib suggested enterohepatic circulation of the metabolite. The AUC from time 0 to 48 h (AUC_0–48_) and AUC from time 0 to 24 h (AUC_0–24_) values of gefitinib and *O*-desmethyl gefitinib were comparable. However, the plasma concentrations and AUC_0–24_ and AUC_0–48_ values of the metabolite showed larger interindividual variability than those of gefitinib, where no metabolite was detectable in one patient (Additional file 1: Table S1).

**Table 2:** Pharmacokinetic parameters in our eighteen patients

Parameter	Value^a^
*Gefitinib*	
AUC_0–48_ (μM h)	9.49 ± 3.5
AUC_0–24_ (μM h)	6.17 ± 1.9
CL/F (L/h)	51.0 ± 25
T_max_ (h)	4.56 ± 1.3
C_max_ (μM)	0.492 ± 0.19
t_1/2_ (h)	24.1 ± 8.6
*O-desmethyl gefitinib*	
AUC_0–48_ (μM h)	10.6 ± 14
AUC_0–24_ (μM h)	5.16 ± 6.7

AUC_0–48_, area under the plasma concentration–time curve from 0 to 48 h; AUC_0–24_, area under the plasma concentration–time curve from 0 to 24 h; CL/F, clearance of the drug from plasma; T_max_, time to the maximum plasma concentration; C_max_, maximum plasma concentration; t_1/2_, the elimination half-life

^a^Mean ± SD

### Toxicities

Toxicity data for all 18 patients observed 30 days after starting gefitinib treatment are presented in Table 3. The most common adverse event was diarrhea (50%), followed by rash (28%), and anorexia and fatigue (22%). Grade 3 diarrhea, an increased AST level, and increased ALT level were observed in one patient each. We did not observe any associations between the pharmacokinetics of gefitinib and toxicities including diarrhea, rash, anorexia, or fatigue (*P* > 0.05) (Additional file 2: Fig. S1) (Additional file 1: Table S1).

**Table 3:** Toxicities observed in our patients during 30 days after initiation of gefitinib treatment

Toxicities	N = 18	
	Number (%)	
	Any	≥ Grade 3
Diarrhea	9 (50)	1 (5.6)
Rash	5 (28)	0 (0)
Anorexia	4 (22)	0 (0)
Fatigue	4 (22)	0 (0)
Nausea	2 (11)	0 (0)
Dry skin	2 (11)	0 (0)
Mucositis	2 (11)	0 (0)
AST increase	1 (5.6)	1 (5.6)
ALT increase	1 (5.6)	1 (5.6)
Vomiting	1 (5.6)	0 (0)
Pharyngitis	1 (5.6)	0 (0)

*AST* Aspartate aminotransferase, *ALT* Alanine aminotransferase

### Effects of genetic polymorphisms on the pharmacokinetics of gefitinib and the metabolite

Next, we examined the associations between the pharmacokinetics of gefitinib and *O*-desmethyl gefitinib, and the polymorphisms in genes encoding factors associated with gefitinib pharmacokinetics. Genotypes and allele frequencies of polymorphisms in the *CYP3A5*, *CYP2D6*, *ABCG2*, *ABCB1*, and *OATP1B1* genes are shown in Table 4. The frequencies of the respective polymorphisms are almost equal to those reported previously in Japanese [20–22]. These allele frequencies were in Hardy–Weinberg equilibrium (*P* > 0.05).

**Table 4:** Genotype and allele frequencies of polymorphisms in the *CYP3A5*, *CYP2D6*, *ABCG2*, *ABCB1*, and *OATP1B1*

	Genotype	Number (%)	Allele frequency	
CYP3A5*3	*1/*1	1 (0.06)	*1	0.22
	*1/*3	6 (0.33)	*3	0.78
	*3/*3	11 (0.61)		
CYP2D6*5 and *10	*1/*1	4 (0.22)	*1	0.47
	*1/*5	1 (0.06)	*5	0.06
	*1/*10	8 (0.44)	*10	0.47
	*5/*10	1 (0.06)		
	*10/*10	4 (0.22)		
*ABCG2* 421C > A	C/C	8 (0.44)	C	0.64
	C/A	7 (0.39)	A	0.36
	A/A	3 (0.17)		
*ABCB1* 1236C > T	C/C	4 (0.22)	C	0.53
	C/T	11 (0.61)	T	0.47
	T/T	3 (0.17)		
*ABCB1* 2677G > T/A	G/G	1 (0.06)	G	0.28
	G/T	7 (0.38)	T	0.61
	G/A	1 (0.06)	A	0.11
	T/T	6 (0.33)		
	T/A	3 (0.17)		
*ABCB1* 3435C > T	C/C	1 (0.06)	C	0.25
	C/T	7 (0.38)	T	0.75
	T/T	10 (0.56)		
*OATP1B1* 388A > G	A/A	3 (0.17)	A	0.36
	A/G	7 (0.38)	G	0.64
	G/G	8 (0.44)		
*OATP1B1* 521 T > C	T/T	13 (0.72)	T	0.83
	T/C	4 (0.22)	C	0.17
	C/C	1 (0.06)		
*OATP1B1* diplotype	*1a/*1a	3 (0.17)		
	*1a/*1b	4 (0.22)		
	*1b/*1b	6 (0.33)		
	*1a/*15	3 (0.17)		
	*1b/*15	1 (0.06)		
	*15/*15	1 (0.06)		

N = 18

Gefitinib metabolism by CYP2D6 to form *O*-desmethyl gefitinib was affected by the *CYP2D6* genotype consisting of *5 and *10 with a clear gene dosage effect (Table 5). Patients possessing the CYP2D6*5/*10 or *10/*10 genotype showed the highest AUC_0-48_ of gefitinib and the lowest AUC_0–48_ of *O*-desmethyl gefitinib, whereas those who had CYP2D6*1/*1 showed the lowest AUC_0–48_ of gefitinib and the highest AUC_0-48_ of *O*-desmethyl gefitinib. The trend of higher AUC_0–48_ value of gefitinib in patients with the *ABCG2* 421AA genotype was observed (Table 5). Comparison of the AUC_0–48_ value of gefitinib between *ABCG2* 421AA, and CC or CA genotypes revealed the higher AUC_0–48_ value in AA genotype (14.0 ± 1.7 µM h, N = 3) than in CC or CA genotype (8.58 ± 3.0 µM h, N = 15) (*P* = 0.0209). However, genotypes or haplotypes in the *CYP3A5*, *ABCG1*, and *OATP1B1* genes did not show clear associations with systemic exposure to gefitinib and *O*-desmethyl gefitinib (Additional file 1: Table S1).

**Table 5:** Associations between polymorphisms and AUC_0–48_ of gefitinib or *O*-desmethyl gefitinib

Polymorphism	Genotype	N	Gefitinib AUC_0–48_ (µM h)^a^	*P*^b^	*O*-desmethyl gefitinib AUC_0–48_ (µM h)^a^	*P*^b^
CYP3A5*3	*1/*1	1	12.1	NA^c^	1.94	NA^c^
	*1/*3	6	8.62 ± 3.5		12.9 ± 12	
	*3/*3	11	9.73 ± 3.6		10.0 ± 16	
CYP2D6*5 and *10	*1/*1	4	5.52 ± 0.61	0.00580	28.2 ± 23	0.0179
	*1/*5 or *1/*10	9	9.53 ± 3.0		6.81 ± 4.8	
	5/*10 or *10/*10	5	12.6 ± 2.2		3.23 ± 2.2	
*ABCG2*421C > A	C/C	8	8.49 ± 3.5	0.0692	16.5 ± 20	0.459
	C/A	7	8.69 ± 2.6		5.14 ± 3.7	
	A/A	3	14.0 ± 1.7		7.43 ± 7.5	
*ABCB1*1236C > T	C/C	4	12.8 ± 2.6	0.0724	7.36 ± 5.9	0.670
	C/T	11	7.99 ± 2.9		13.4 ± 18	
	T/T	3	10.6 ± 3.7		4.57 ± 1.6	
*ABCB1*2677G > T/A	G/G	1	15.3	NA^c^	4.36	NA^c^
	G/T	7	8.59 ± 3.9		20 ± 20	
	G/A	1	7.55		6.79	
	T/T	6	9.33 ± 2.8		20 ± 20	
	T/A	3	10.6 ± 3.7		4.57 ± 1.6	
*ABCB1* 3435C > T	C/C	1	12.2	NA^c^	0.00	NA^c^
	C/T	7	9.42 ± 4.2		20.0 ± 20	
	T/T	10	9.27 ± 3.2		5.03 ± 2.9	
*ABCB1* diplotype	Any/Any	6	11.8 ± 2.9	0.0492	6.04 ± 5.4	0.640
	TTT/Any	12	8.33 ± 3.2		12.8 ± 17	
*OATP1B1*388A > G	A/A	3	8.89 ± 3.1	0.474	5.10 ± 3	0.319
	A/G	7	10.8 ± 2.9		11.2 ± 20	
	G/G	8	8.55 ± 4.0		12.1 ± 11	
*OATP1B1*521T > C	T/T	13	9.88 ± 3.7	NA^c^	8.87 ± 9.3	NA^c^
	T/C	4	8.7 ± 3.3		17 ± 27	
	C/C	1	7.55		6.79	
*OATP1B1*diplotype	*1a/*1a, *1a/*1b, or *1b/*1b	13	9.88 ± 3.7	NA^c^	8.87 ± 9.3	NA^c^
	*1a/*15 or *1b/*15	4	8.70 ± 3.3		17.0 ± 27	
	15/*15	1	7.55		6.79	

N = 18

*AUC* area under the plasma concentration–time curve from time 0 to 48 h

^a^Mean ± SD

^b^Wilcoxon or Kruskal–Wallis test

^c^Not analyzed because of the small number of patients in a genotype (N < 3)

## Discussion

Commensurate with the aging population in Japan, the number of patients with cancer is also increasing [11]. Nevertheless, elderly patients with cancer are generally excluded from clinical trials, which results in the lack of data on pharmacokinetics, safety, and efficacy of anticancer drugs in this population.

In this prospective study, we examined the pharmacokinetics of gefitinib and its major metabolite *O*-desmethyl gefitinib for the first time in elderly patients with NSCLC (Table 2). To date, the AUC_0–24_ value of gefitinib in younger Japanese patients was obtained in a phase I study [23]. The AUC_0–24_ value of gefitinib observed in patients with a median age of 61 years (range, 41–73 years) at a dose of 225 mg was 1986 ng h/mL which is equivalent to 4.44 µM h. Because the relationship between the gefitinib dose and its AUC were reported to be linear [23, 24], the AUC_0–24_ value of gefitinib at a dose of 250 mg in younger patients was estimated to be 4.9 µM h, which is somewhat lower than the 6.17 ± 1.9 µM h obtained in elderly patients. A population pharmacokinetic analysis revealed that the CL/F value of gefitinib was negatively correlated with age [25]. Taking these results into account, systemic exposure to gefitinib in elderly patients may be slightly higher than that in younger patients.

We evaluated gefitinib-induced toxicities in elderly patients with advanced NSCLC for 30 days after the initiation of gefitinib treatment. In a phase I study of gefitinib performed in younger Japanese patients with cancer, toxicities were evaluated for a similar period to that in the present study [23]. Another phase II study of gefitinib for neoadjuvant therapy in younger Chinese patients with resectable early-stage NSCLC also examined the gefitinib-induced toxicities for almost a comparable period to that in our study [26]. Profiles, frequencies, and grades of gefitinib-induced toxicities observed in our study of elderly patients with NSCLC were roughly similar to those observed in both previous studies performed in younger patients. For example, two patients treated with 225 mg of gefitinib had a grade 3 AST level increase and ALT level increase, respectively [23]; no grade 3/4 adverse event was observed in a neoadjuvant setting of gefitinib therapy [26]. According to a phase II study performed in Japan with elderly patients with advanced NSCLC harboring *EGFR* mutations (NEJ003), where the toxicities were evaluated beyond 30 days, first-line treatment with gefitinib was concluded to be tolerable [10]. These results suggest that first-line treatment of elderly patients with advanced NSCLC is preferable to standard chemotherapy for this population. It seems likely that the impact of the slightly higher exposure to gefitinib in elderly patients than in younger patients (Table 2) might be small considering the tolerability for gefitinib treatment among our patients.

Pharmacogenetic analyses revealed that gefitinib metabolism by CYP2D6 to form *O*-desmethyl gefitinib was affected by the *CYP2D6* genotype consisting of *5 and *10 with a clear gene dosage effect, which is consistent with the previous results obtained for Japanese patients with advanced NSCLC [27]. *ABCG2* 421C > A was reported to be associated with gefitinib pharmacokinetics and toxicities [12]. Consistent with these previous results, the AUC_0–48_ value of gefitinib was higher in patients harboring the *ABCG2* 421AA genotype than in those with the *ABCG2* 421CC or CA genotype in this study.

The present study had several limitations. First, the sample size was small, especially for evaluating the gefitinib-induced toxicities. Second, we did not perform a validation study for the present findings. Therefore, further additional validation with a large numbers of patients is necessary to definitively confirm our results. Third, adherence to gefitinib therapy for the first 30 days was not fully monitored: compliance with gefitinib treatment during the 1-week hospitalization period from the beginning of treatment was monitored by ward nursing staff. However, compliance with the gefitinib treatment was not completely monitored after discharge (Additional file 2: Table S1).

## Conclusions

We examined the pharmacokinetics of gefitinib and its major metabolite *O*-desmethyl gefitinib for the first time in elderly patients with NSCLC.

## Supplementary Information

**Additional file 1**. **Table S1.** Raw data generated or analyzed during this study.

**Additional file 2**. **Fig. S1.** Associations between the pharmacokinetics of gefitinib and toxicities.

## Data Availability

All data generated or analyzed during this study are included in this published article and its supplementary information files.

## Abbreviations

ALT: Alanine transaminase
AST: Aspartate transaminase
AUC: Area under the plasma concentration–time curve
AUC_0-48_: AUC from time 0 to 48 h
AUC_0-24_: AUC from time 0 to 24 h
CL/F: Oral clearance
C_max_: Maximum plasma concentration
EGFR: Epidermal growth factor receptor
NSCLC: Non-small cell lung cancer
PCR: Polymerase chain reaction
OATP1B1: Organic anion transporting polypeptide
t_1/2_: Elimination half-life
TKI: Tyrosine kinase inhibitor
T_max_: Times to the maximum plasma concentration
