# Supplementary material for: Pharmacokinetics of gefitinib in elderly patients with EGFR-mutated advanced non-small cell lung cancer: a prospective study
Source: BMC Pulm Med. 2022 Nov 30;22:454. doi: 10.1186/s12890-022-02249-8 (PMC9710131; doi:10.1186/s12890-022-02249-8)
Supplement: Supplementary file 1 — Additional file 1. Table S1. Raw data generated or analyzed during this study. [file 12890_2022_2249_MOESM1_ESM.pdf]

Supplementary Table 1. Raw data generated or analysed during this study

Patient	Patient characteristics (baseline)								
	Age	Performance status	Serum albumin (g/dL)	Total bilirubin (mg/dL)	Serum creatinin (mg/dL)	eGFR (mL/min)	*EGFR* muttion type		Diarrhea
1	76	2	3.3	0.4	0.83	50.7	Ex19del		1
2	76	1	4.5	1.0	0.57	76.5	L858R		0
3	78	2	3.0	0.7	0.72	79.6	L858R		1
4	76	1	4.2	1.0	0.71	60.2	Ex19del		0
5	76	3	3.6	0.8	1.14	48.5	Ex19del		3
6	89	2	3.6	0.5	0.92	58.6	Ex19del		2
7	80	3	1.6	0.5	0.80	70.4	L858R		0
8	84	1	4.4	0.5	0.42	103.8	Ex19del		1
9	85	1	3.7	0.9	0.73	56.5	Ex19del		0
10	86	3	2.6	0.8	0.38	115.1	Ex19del		1
11	75	0	3.9	0.6	0.64	67.7	L858R		1
12	81	1	3.9	0.6	0.81	51.1	L858R		1
13	77	2	2.7	0.5	0.65	89.3	L858R		0
14	81	1	3.6	0.7	0.77	54.1	L858R		0
15	85	1	3.9	0.6	0.71	58.5	L858R		0
16	79	2	3.6	0.5	0.56	77.1	Ex19del		0
17	85	1	3.6	0.6	0.91	60.1	L858R		0
18	81	1	3.8	0.6	0.81	51.1	L858R		1

Adverse events of gefitinib (grade)												
Rash	Anorexia	Fatigue	Nausea	Dry skin	Mucositis	ALT increase	AST increase	Vomiting	Pharyngitis		*ABCG2* 421C>A	*CYP3A5*
0	2	2	0	0	0	0	0	0	0		C/C	*1/*3
0	0	0	0	0	0	0	0	0	0		C/C	*3/*3
1	1	1	0	0	0	0	0	0	0		C/C	*3/*3
0	0	0	0	0	0	0	0	0	0		C/C	*3/*3
0	0	0	0	0	0	0	0	0	0		A/C	*1/*3
0	0	0	1	0	0	0	0	0	0		A/C	*1/*3
0	0	0	0	0	0	0	0	0	0		C/C	*3/*3
1	0	1	0	0	0	0	0	0	0		A/C	*3/*3
0	0	0	0	1	0	0	0	0	1		A/A	*1/*3
1	0	0	0	0	0	0	0	0	0		C/C	*3/*3
1	0	0	0	0	1	0	0	0	0		A/C	*1/*3
0	2	2	0	0	0	0	0	2	0		A/A	*3/*3
0	0	0	0	1	0	3	3	0	0		C/C	*1/*3
0	0	0	0	0	0	0	0	0	0		A/C	*3/*3
0	0	0	0	0	2	0	0	0	0		A/C	*3/*3
0	0	0	0	0	0	0	0	0	0		A/A	*1/*1
1	0	0	1	0	0	0	0	0	0		A/C	*3/*3
0	1	0	0	0	0	0	0	0	0		C/C	*3/*3

Genotypes										
CYP2D6	*ABCB1* 1236 C>T	*ABCB1* 2677 G>T/A	*ABCB1* 3435 C>T	*OATP1B1* 521 T>C	*OATP1B1* 388A>G	*OATP1B1* diplotype		0 h	1 h	2 h
*1/*1	C/T	G/T	C/T	T/T	G/G	*1b/*1b		0	0.07044693	0.233541409
*1/*1	C/T	T/T	T/T	T/T	A/A	*1a/*1a		0	0	0.124736824
*1/*1	C/T	G/T	C/T	T/C	A/G	*1a/*15		0	0	0
*1/*10	C/T	G/T	C/T	T/T	G/G	*1b/*1b		0	0	0.136838419
*1/*10	C/T	T/T	T/T	T/T	A/A	*1a/*1a		0	0	0
*1/*1	C/T	G/T	T/T	T/T	G/G	*1b/*1b		0	0	0.04783806
*10/*10	T/T	T/A	T/T	T/T	A/G	*1a/*1b		0	0	0.203423595
*1/*10	C/T	G/A	C/T	C/C	G/G	*15/*15		0	0	0.046346888
*1/*10	C/C	G/T	C/T	T/T	G/G	*1b/*1b		0	0.07746181	0.163152529
*1/*10	T/T	T/A	T/T	T/C	A/G	*1a/*15		0	0.48431534	0.772738914
*10/*10	C/C	T/T	T/T	T/T	A/G	*1a/*1b		0	0	0.236410765
*5/*10	C/C	G/G	C/T	T/T	G/G	*1b/*1b		0	0	0
*1/*10	T/T	T/A	T/T	T/C	G/G	*1b/*15		0	0.04852707	0.187143028
*1/*10	C/T	T/T	T/T	T/T	G/G	*1b/*1b		0	0	0.130134733
*10/*10	C/C	G/T	C/T	T/T	A/G	*1a/*1b		0	0	0.314785187
*1/*10	C/T	T/T	T/T	T/T	A/A	*1a/*1a		0	0	0.079425779
*10/*10	C/T	G/T	C/C	T/C	A/G	*1a/*15		0	0.08009066	0.205458628
*1/*5	C/T	T/T	T/T	T/T	A/G	*1a/*1b		0	0	0.065861825

Plasma concentrations (μM)												
Gefitinib					*O*-desmethyl gefitinib							
4 h	6 h	8 h	24 h	48 h	0 h	1 h	2 h	4 h	6 h	8 h	24 h	48 h
0.30974199	0.307127	0.25624	0.1012236	0.04016554	0	0.18804536	0.52413323	0.43677241	0.327865993	0.69762884	0.9194947	0.7532245
0.2708922	0.207818	0.17822	0.1074535	0.08972165	0	0	0.30349915	0.2785483	0.167864378	0.156201702	0.14626192	0.18042533
0.20698022	0.215798	0.19028	0.1140374	0.04046408	0	0	0	1.38842467	1.321471355	1.495963022	1.11372945	1.33590068
0.29675024	0.291801	0.23985	0.1292241	0.05498788	0	0.07946477	0.23106169	0.19625568	0.142867855	0.127028193	0.43700001	0.22950208
0.27591522	0.342726	0.29973	0.1898838	0.09286651	0	0	0	0.07317889	0.060751089	0.056597627	0.18515807	0.09740394
0.29924297	0.261968	0.20242	0.0879674	0.04051944	0	0	0.0534492	0.33337937	0.222044546	0.139800043	0.33027169	0.22414952
0.4531762	0.52705	0.48825	0.2501432	0.22747045	0	0	0.0599757	0.0568621	0.053991975	0.052603004	0.05662245	0.06333021
0.48011798	0.478962	0.24602	0.1357387	0.06954898	0	0	0.06299437	0.16906933	0.184422499	0.125394802	0.17089734	0.12329204
0.76577314	0.520059	0.48002	0.2624251	0.19697144	0	0	0.09382872	0.51432666	0.422234379	0.445474613	0.35460019	0.24672206
0.62710084	0.430124	0.30969	0.1334961	0.13900354	0	0.13358213	0.15229314	0.15368606	0.164764624	0.164189438	0.08729903	0.12144656
0.48495624	0.479091	0.36623	0.1967835	0.10452743	0		0.08446319	0.1005898	0.075471958	0.058353141	0.10104466	0
0.05717423	0.322159	0.47675	0.3652422	0.25846844	0	0	0	0	0.055087199	0.057717449	0.11839687	0.11397648
0.52311243	0.288705	0.23709	0.1127784	0.06176428	0	0.06927277	0.15218419	0.13439788	0.081206752	0.069106664	0.11012647	0.15290393
0.42160772	0.35222	0.23734	0.106989	0.05111321	0	0	0.06766035	0.12329823	0.13203167	0.090860427	0.07541199	0
0.75998326	0.447737	0.37683	0.1703046	0.10397962	0	0	0.19261802	0.22955964	0.354268186	0.349955205	0.09485126	0
0.40837035	0.480957	0.43397	0.2244899	0.16599947	0	0.05565102	0.05591876	0.06303912	0.060467809	0.054312272	0.05344126	0
0.6322078	0.459913	0.42875	0.2481688	0.09910281	0	0	0	0	0	0	0	0
0.79348652	0.658219	0.45109	0.1948552	0.14565316	0	0	0	0.09738245	0.103043866	0.114937672	0.05403741	0.09839564

Pharmacokinetic parameters								
Gefitinib								*O* -desmeth
AUC_{0-48} (μM·h)	AUC_{0-24} (μM·h)	Cmax (h)	tmax (h)	t1/2 (h)	kel (1/h)	CL (L/h)	V (L)	AUC_{0-48} (μM·h)
6.148207	4.5796558	0.30974199	4	14.278229	0.048545739	80.431541	1656.8198	36.136633
5.9167594	3.5570503	0.2708922	4	35.90582	0.019304592	52.951203	2742.9331	7.8335611
5.1046491	3.4180031	0.21579793	6	17.453856	0.039713127	91.72931	2309.7982	57.038149
6.5676436	4.4824427	0.29675024	4	18.873415	0.036726113	69.362422	1888.6404	13.47207
8.6831392	5.427749	0.34272612	6	23.630416	0.029332839	47.210351	1609.4709	5.5368442
4.8929939	3.5905683	0.29924297	4	14.732986	0.047047299	100.94392	2145.5837	11.648549
14.278467	8.5514111	0.52704986	6	38.723249	0.017900026	20.729041	1158.0453	2.7074396
7.550755	5.1751719	0.48011798	4	22.151778	0.031290814	57.236875	1829.191	6.7926622
14.598188	9.1229364	0.76577314	4	29.743778	0.023303939	24.268486	1041.3899	15.967069
10.663679	7.393684	0.77273891	2	22.231999	0.031177906	36.99227	1186.4899	5.6156334
10.508322	7.008552	0.48495625	4	22.394395	0.030951816	40.28685	1301.5989	3.0229557
15.342647	7.931815	0.47675331	8	45.497751	0.015234757	17.314407	1136.5069	4.3649503
6.8755667	4.8421176	0.52311243	4	19.32997	0.035858679	65.061621	1814.3898	5.3829537
6.4035595	4.5881409	0.42160772	4	18.344421	0.037785176	72.122098	1908.7406	2.9317391
10.621884	7.3956575	0.75998326	4	20.215662	0.034287633	40.968319	1194.8425	6.071269
12.06644	7.4158235	0.48095732	6	29.979411	0.023120774	29.065618	1257.1213	1.9440319
12.173482	8.2761256	0.6322078	4	18.995912	0.036489282	37.570288	1029.6253	0
12.377669	8.3201667	0.79348652	4	20.678043	0.033520928	33.451315	997.92332	3.6360785

yl gefitinib
AUC_{0-24} (μM·h)
16.130287
3.9133141
27.642587
5.739481
2.2580689
5.0775557
1.2680077
3.2934214
8.829292
3.1106863
1.8104197
1.5768064
2.2265889
2.0267952
4.9330539
1.3027368
0
1.806882
