# Supplementary material for: Pharmacokinetics of gefitinib in elderly patients with EGFR-mutated advanced non-small cell lung cancer: a prospective study
Source: BMC Pulm Med. 2022 Nov 30;22:454. doi: 10.1186/s12890-022-02249-8 (PMC9710131; doi:10.1186/s12890-022-02249-8)
Supplement: Supplementary file 2 — Additional file 2. Fig. S1. Associations between the pharmacokinetics of gefitinib and toxicities. [file 12890_2022_2249_MOESM2_ESM.pptx]

## Slide 1
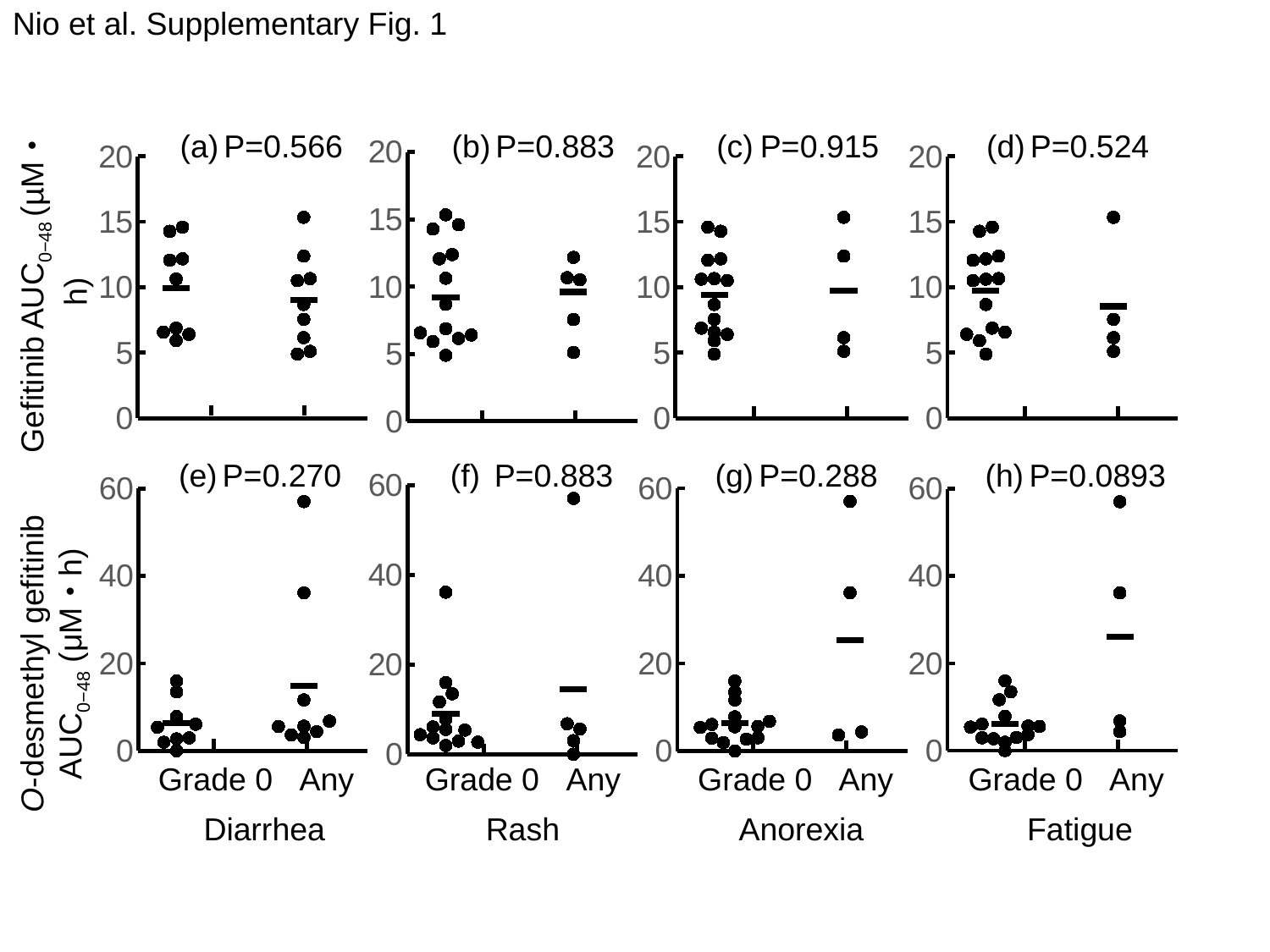

Nio et al. Supplementary Fig. 1
Gefitinib AUC0−48 (µM･h)
(a)
P=0.566
(b)
P=0.883
(c)
P=0.915
(d)
P=0.524
### Chart
| Category | gefitinib
AUCall (uMh) |
|---|---|
### Chart
| Category | gefitinib
AUCall (uMh) |
|---|---|
### Chart
| Category | gefitinib
AUCall (uMh) |
|---|---|
### Chart
| Category | gefitinib
AUCall (uMh) |
|---|---|(e)
P=0.270
(f)
P=0.883
(g)
P=0.288
(h)
P=0.0893
### Chart
| Category | O-desmethyl gefitinib
AUCall (uMh) |
|---|---|
### Chart
| Category | O-desmethyl gefitinib
AUCall (uMh) |
|---|---|
### Chart
| Category | O-desmethyl gefitinib
AUCall (uMh) |
|---|---|
### Chart
| Category | O-desmethyl gefitinib
AUCall (uMh) |
|---|---|O-desmethyl gefitinib AUC0−48 (μM･h)
Grade 0
Any
Grade 0
Any
Grade 0
Any
Grade 0
Any
Diarrhea
Rash
Anorexia
Fatigue
